# Population normative data for the 10/66 Dementia Research Group cognitive test battery from Latin America, India and China: a cross-sectional survey

**DOI:** 10.1186/1471-2377-9-48

**Published:** 2009-08-26

**Authors:** Ana Luisa Sosa, Emiliano Albanese, Martin Prince, Daisy Acosta, Cleusa P Ferri, Mariella Guerra, Yueqin Huang, KS Jacob, Juan Llibre de Rodriguez, Aquiles Salas, Fang Yang, Ciro Gaona, AT Joteeshwaran, Guillermina Rodriguez, Gabriela Rojas de la Torre, Joseph D Williams, Robert Stewart

**Affiliations:** 1 National Institute of Neurology and Neurosurgery of Mexico, Mexico City, Mexico; 2 King's College London (Institute of Psychiatry), London, UK; 3 Universidad Nacional Pedro Henriquez Ureña (UNPHU), Santo Domingo, Dominican Republic; 4 Instituto de la Memoria y Desórdenes Relacionados, Universidad Peruana Cayetano Heredia,. Lima, Peru; 5 Institute of Mental Health, Peking University, Beijing, PR China; 6 Christian Medical College, Vellore, India; 7 Facultad de Medicina Finley-Albarran, Medical University of Havana, Cuba; 8 Caracas University Hospital Faculty of Medicine, Universidad Central de Venezuela, Caracas, Venezuela; 9 Clínica Loira, Caracas, Venezuela; 10 Community Health Services, VHS, Chennai, India; 11 Dirección General de Salud Publica Ministerio de Protección Social (6th district), Santo Domingo, Dominican Republic

## Abstract

**Background:**

1) To report site-specific normative values by age, sex and educational level for four components of the 10/66 Dementia Research Group cognitive test battery; 2) to estimate the main and interactive effects of age, sex, and educational level by site; and 3) to investigate the effect of site by region and by rural or urban location.

**Methods:**

Population-based cross-sectional one phase catchment area surveys were conducted in Cuba, Dominican Republic, Venezuela, Peru, Mexico, China and India. The protocol included the administration of the Community Screening Instrument for Dementia (CSI 'D', generating the COGSCORE measure of global function), and the Consortium to Establish a Registry for Alzheimer's Disease (CERAD) verbal fluency (VF), word list memory (WLM, immediate recall) and recall (WLR, delayed recall) tests. Only those free of dementia were included in the analysis.

**Results:**

Older people, and those with less education performed worse on all four tests. The effect of sex was much smaller and less consistent. There was a considerable effect of site after accounting for compositional differences in age, education and sex. Much of this was accounted for by the effect of region with Chinese participants performing better, and Indian participants worse, than those from Latin America. The effect of region was more prominent for VF and WLM than for COGSCORE and WLR.

**Conclusion:**

Cognitive assessment is a basic element for dementia diagnosis. Age- and education-specific norms are required for this purpose, while the effect of gender can probably be ignored. The basis of cultural effects is poorly understood, but our findings serve to emphasise that normative data may not be safely generalised from one population to another with quite different characteristics. The minimal effects of region on COGSCORE and WLR are reassuring with respect to the cross-cultural validity of the 10/66 dementia diagnosis, which uses only these elements of the 10/66 battery.

## Background

Rapid demographic ageing around the world has important implications for health and social care. Cognitive decline and dementia have a high individual impact and are strongly age-associated [1], so that their overall prevalence and societal impact is increasing rapidly. A recent consensus report estimated that the number of people with dementia in the world will increase from 24 million to 82 million from 2000 to 2040 [2]. This increase will be particularly marked in low and middle income countries where epidemiological research into the aetiology and impact of dementia and cognitive decline is limited. The 10/66 Dementia Research Programme was set up to facilitate research in these regions and to provide data that can be used for public health and service planning [1]. Cognitive tests covering multiple domains are an essential component of a definitive dementia diagnostic assessment: for the purposes of establishing the criterion of decline in at least two domains of cognitive function, including memory [3]. Normative data are urgently required, given the influence of both education and culture on cognitive test performance [4,5].

The data presented in this paper were drawn from the 10/66 Dementia Research Group's cross-sectional surveys of older people carried out in seven urban and four rural sites in five Latin American countries, China and India. The primary objective was to generate site-specific norms for the cognitive test battery used in the 10/66 studies comprising tests of general cognitive function, verbal fluency and immediate and delayed verbal recall. Further objectives were: a) to assess the independent influences of age, educational level and gender and their homogeneity across sites, and b) to assess the extent to which variance attributable to site could be attributed to the effects of region and/or rural versus urban residence.

## Method

### Study design

The design of the 10/66 Dementia Research Group (DRG) baseline population-based studies has been described in detail [6]. Briefly, cross-sectional surveys were carried out, approaching all residents aged 65 and over within purposively selected geographically-defined catchment areas at each site. No over-sampling strategy was applied (e.g. with respect to age groups). Affluent districts were intentionally avoided. A target sample of 2000 persons aged 65 years and over, per country (3,000 in Cuba) was identified by means of door knocking the catchment areas. Peru, Mexico, China and India recruited both from rural and urban sites. Interviews followed a comprehensive one-phase design where all participants received a full assessment including: cognitive and mental health evaluation, an informant interview, a physical and neurological examination, blood assays and genotyping, in addition to questionnaire measures of environmental and behavioural risk exposures, sociodemographic and socioeconomic status, and physical health status. Disability, health service utilisation, care arrangements and impact of providing care were also evaluated.

### Measurements

For this analysis we considered the following socio-demographic measures as independent variables: participants' age divided into four groups (65-69 years, 70-74 years, 75-79 years, 80 years and over), sex, and education level divided into five groups (none, some (but did not complete primary), completed primary, completed secondary, and tertiary). Age of participants was formally established during interview from stated age, official documentation, informant report and, in the case of discrepancy, age according to an event calendar.

The 10/66 cognitive assessment battery was drawn principally from the Community Screening Instrument for Dementia (CSI 'D') developed by the Ibadan-Indianapolis study group [7] specifically for use in cross-cultural research, and in low education settings, and from the Consortium to Establish a Registry for Alzheimer's Disease (CERAD) [8]. As such, components of the battery have been very widely used in other population and clinical research. In our large multi-site pilot study [9] we developed and validated a culture- and education-fair algorithm for dementia diagnosis across a wide variety of low and middle income country settings, comprising components of the cognitive test battery in combination with the Geriatric Mental State and the informant section of the CSI 'D'

The analysis described here focussed on the four main tests included in the 10/66 cognitive test battery:

1) Global cognitive function: The Community Screening Instrument for Dementia (CSI 'D') [7] includes a 32 item cognitive test assessing orientation, comprehension, memory, naming and language expression, which is used to generate a global cognitive score (COGSCORE). The CSI 'D' was from the outset intended to be used across cultures with the minimum of necessary adaptation. It was developed and first validated among Cree American Indians [7,10], further validated and used in population-based research (The US-Nigeria Study) among Nigerians in Ibadan and African-Americans in Indianapolis [11], and has also been validated among white Canadians in Winnipeg [12], and in Jamaica in conjunction with the CERAD battery [13]. The CSI 'D' test score distributions among those with dementia and controls, and the degree of discrimination provided were remarkably consistent across the aforementioned cultural settings [12].

2) Memory: The 10/66 battery includes two elements of the CERAD 10 word list learning test: world list memory (WLM) and word list recall (WLR), testing immediate and delayed recall respectively. WLR has been reported to be of particular value in distinguishing early dementia from normal aging [14]. WLM and WLR are taken from the adapted CERAD ten word list learning task used in the Indo-US Ballabgarh dementia study [15]. Six words; butter, arm, letter, queen, ticket, and grass; were taken from the original CERAD battery English language list [16]. Pole, shore, cabin, and engine were replaced with corner, stone, book and stick, which were deemed more cross-culturally applicable. In the learning phase, the list is read out to the participant from a green card, who is then asked to recall straight away the words that they remember. This process is repeated three times, giving a WLM score out of 30. In the 10/66 protocol, approximately five minutes later, after a series of unrelated CSI 'D' questions (name registration, object naming, object function, repetition) the participant is again asked to recall the 10 words with prompting that they were read from a green card, giving a WLR score out of 10.

3) Verbal fluency (VF): the animal naming verbal fluency task [7] from the CERAD is administered as part of the CSI 'D', however it is accorded very little weight within the algorithm for calculating the total CSI 'D' score. In the version of the test used in CSI 'D', after a brief practice naming items from another category (clothing), participants are encouraged to name as many different animals as they can in the space of one minute. The instructions read out to the participant stipulate: 'think of any kinds of animal in the air, on land, in the water, in the forest, all the different animals'. If the participant stops before the allotted time has elapsed they are encouraged to continue. The score is one point for each valid name. In the computation of the CSI 'D' cognitive test (COGSCORE) the VF score is divided by 23. These weighted scores generally range between 0 and 1, the same as for a single CSI 'D' orientation item.

The CERAD neuropsychological battery has been adapted for use in India [15], Korea [17], Brazil [18], Nigeria [16] and Jamaica [13], and norms have been provided for black and white persons in the USA, both with dementia [19], and among the general population [20]. While education effects are prominent, cultural or ethnic differences have been less evident [13,17]. CERAD battery components have been found to distinguish reliably between those with dementia and controls across cultures [13,15].

### Ethical considerations

The study was carried out in compliance with the Helsinki Declaration and all participants provided informed consent. The study was considered and approved by the appropriate Research Ethics Committee at King's College London (Institute of Psychiatry and South London & Maudsley NHS Trust (references 076/03 and 209/01) after approvals at all participating sites (Ministerio de Salud Pública in Cuba, Bioethics National Committee for Research in Dominican Republic, Instituto de la Memoria in Peru, Medical Ethics Committee of Peking University the Sixth Hospital in China, Institutional Review Board of Christian Medical College in Vellore, Institutional Review Board of Voluntary Health Services in Chennai, Instituto Nacional de Neurologia y Neurocirugia in Mexico, Universidad Central de Venezuela).

### Statistical analysis

For this analysis, all participants who had received a diagnosis of dementia according to either DSM-IV [3] or 10/66 dementia criteria were excluded [9]. Participants' age, sex and education data were described by site. Means and standard deviations (SD) for each of the four cognitive tests were calculated by age, sex and education for each of the eleven sites. General linear models were used to determine the unadjusted and independent effects of age, sex and education on cognitive test scores across sites. We then tested formally for effect modification by extending the models used to estimate the main effects of age, education and sex to include site by age, site by education and site by sex interaction terms. Finally, we estimated the proportion of the variance (eta^2^) in each cognitive test accounted for by age, education, gender and site. We further sought to investigate the variance accounted for by site by substituting this variable with two further variables sub-classifying sites into region (Latin America versus (a) China, and (b) India) and rural or urban location. The effect of region (controlling for age, education, gender and rural/urban location) is summarised as adjusted means and mean differences with 95% confidence intervals for the two contrasts: China versus Latin America and India versus Latin America. All analyses were carried out using STATA 9.2 (StataCorp. 2007. *Stata Statistical Software: Release *10. College Station, TX: StataCorp LP) on release 1_5 of the 10/66 baseline dataset.

## Results

Response rates for the sites were as follows: Cuba 94%, Dominican Republic 95%, urban Peru 80%, rural Peru 88%, Venezuela 80%, urban Mexico 84%, rural Mexico 86%, urban China 74%, rural China 96%, urban India 72%, rural India 98%. In all, 14,967 participants were fully evaluated of whom 1318 met criteria for dementia [21] and were excluded from further analysis, leaving a total of 13,649 participants, free of dementia (Table 1).

**Table 1:** Sociodemographic characteristics of participants by site

**Region**	**Latin America**							**China**		**India**	
**Country**	**Cuba**	**DR**	**Peru**		**Venezuela**	**Mexico**					
Rural or urban site	Urban	Urban	Urban	Rural	Urban	Urban	Rural	Urban	Rural	Urban	Rural

**Total **(n)	2,621	1,769	1,251	516	1,826	910	913	1,076	946	930	891
**Age in years **(MV)	7	0	1	0	23	1	0	0	0	4	0
65 - 69 years %	28.2	29.0	29.2	33.5	44.1	26.7	32.2	28.7	39.9	42.9	34.8
70 - 74 years %	28.3	27.3	27.3	25.8	24.9	34.4	26.2	32.5	30.1	32.1	34.9
75 - 79 years %	22.3	19.5	21.9	18.2	17.8	20.2	22.1	21.9	19.3	14.6	17.7
80+ years %	21.3	24.3	21.6	22.5	13.1	18.7	19.5	16.8	11.0	10.5	12.6
**Females **(MV)	0	2	0	0	60	0	0	0	0	15	0
%	64.4	65.4	64.1	52.5	63.5	65.6	59.9	56.6	55.3	57.3	52.3
**Education **(MV)	8	19	8	8	63	0	0	0	0	2	0
No education %	2.1	17.8	2.5	14.0	7.3	19.6	31.0	19.2	57.2	41.3	63.6
Some education (did not complete primary) %	20.9	51.8	6.4	25.6	22.1	35.7	52.3	12.8	11.5	24.2	21.0
Completed primary %	33.0	19.1	31.8	49.6	49.4	24.2	12.8	26.5	26.3	20.8	12.4
Completed secondary %	26.0	7.1	36.1	6.6	14.3	10.4	2.4	29.4	4.4	9.1	2.8
Completed tertiary %	17.9	3.7	22.8	3.1	5.0	10.1	1.5	12.1	0.5	4.4	0.2

DR = Dominican Republic; MV = number of records with missing information on the variable in question.

All age groups were well represented. The Venezuelan, rural Chinese and Indian samples had a younger age distribution than other sites. The female/male ratio exceeded 1 in all sites, but with a less striking preponderance of women in rural Peru, China and India. Educational level showed considerable variation across sites, highest in urban Latin America sites (other than the Dominican Republic), and lowest in rural China and in India. Within countries, educational levels were consistently higher in urban compared with rural sites.

Tables 2, 3, 4 and 5 present normative data: stratified means and standard deviations for the four cognitive outcomes. Older age and lower levels of education were consistently associated with poorer cognitive test performance on scores for all four tests, across all sites. The effect of sex on cognitive test performance was smaller and more variable, both between tests and between sites. Men tended to perform marginally better than women on the COGSCORE, and on VF. For WLM and WLR, women performed better than men in Latin American sites, but there was no gender difference in China and India.

**Table 2:** Mean (SD) scores for global cognitive function (CSI 'D' COGSCORE) by demographic status and site

	**Country**										
	
	**Cuba**	**DR**	**Peru**		**Venezuela**	**Mexico**		**China**		**India**	
	Urban	Urban	Urban	Rural	Urban	Urban	Rural	Urban	Rural	Urban	Rural
**Age**											
65 - 69	31.1 (1.9)	30.4 (2.3)	31.8 (1.3)	30.6 (2.9)	31.1 (1.8)	30.8 (1.7)	29.8 (2.1)	31.7 (1.2)	31.0 (3.9)	29.2 (3.0)	27.9 (3.2)
70 - 74	30.8 (1.9)	30.2 (2.1)	31.3 (2.2)	30.4 (1.8)	30.6 (2.2)	30.1 (2.8)	29.3 (1.9)	31.4 (2.0)	30.7 (2.6)	28.3 (3.7)	27.3 (4.3)
75 - 79	30.3 (2.1)	29.9 (2.1)	31.1 (2.4)	29.7 (2.7)	30.1 (2.5)	29.5 (2.8)	28.5 (3.4)	31.4 (1.5)	29.6 (4.3)	28.5 (4.0)	27.0 (3.6)
80+	29.5 (2.6)	28.7 (2.8)	29.6 (3.4)	28.9 (3.4)	29.0 (2.8)	29.3 (2.4)	28.3 (2.6)	30.9 (2.8)	29.1 (3.7)	27.6 (3.4)	25.3 (7.3)
Crude **β** (95% C.I.)	-0.5 (-0.6 to -0.5)	-0.5 (-0.6 to -0.4)	-0.6 (-0.8 to -0.5)	-0.6 (-0.8 to -0.4)	-0.6 (-0.7 to-0.5)	-0.5 (-0.7 to -0.4)	-0.5 (-0.7 to -0.4)	-0.2 (-0.3 to -0.1)	-0.7 (-0.9 to -0.4)	-0.8 (-0.7 to -0.3)	-0.8 (-1.0 to -0.5)
Adj. **β^1^** (95% C.I.)	-0.4 (-0.4 to -0.3)	-0.5 (-0.6 to -0.4)	-0.6 (-0.7 to -0.4)	-0.5 (-0.7 to -0.3)	-0.5 (-0.6 to -0.4)	-0.4 (-0.5 to -0.2)	-0.5 (-0.6 to -0.3)	-0.2 (-0.3 to -0.9)	-0.6 (-0.8 to -0.3)	-0.5 (-0.7 to -0.3)	-0.8 (-1.1 to -0.5)
**Sex**											
Females	30.4 (2.3)	29.6 (2.6)	31.0 (2.2)	29.9 (2.7)	30.4 (1.89)	29.9 (2.5)	29.0 (2.5)	31.2 (2.3)	30.2 (3.1)	27.9 (3.4)	26.0 (5.3)
Males	30.7 (2.1)	30.2 (2.01)	31.1 (2.9)	30.2 (3.0)	30.7 (2.54)	30.2 (2.5)	29.2 (2.7)	31.6 (1.2)	30.7 (4.3)	29.7 (3.1)	28.5 (2.52)
Crude **β** (95% C.I.)	0.3 (0.1 to 0.5)	0.6 (0.3 to 0.8)	0.0 (-0.2 to 0.3)	0.3 (-0.2 to 0.7)	0.3 (0.1 to 0.5)	0.4 (0.0 to 0.7)	0.2 (-0.1 to 0.5)	0.4 (0.2 to 0.7)	0.5 (0.0 to 0.9)	1.9 (1.4 to 2.3)	2.6 (2.0 to 3.1)
Adj. **β^2^** (95% C.I.)	0.1 (-0.1 to 0.2)	0.4 (0.1 to 0.6)	0.0 (-0.2 to 0.3)	0.1 (-0.4 to 0.6)	0.2 (-0.0 to 0.4)	0.4 (0.0 to 0.7)	0.2 (-0.1 to 0.6)	0.2 (-0.1 to 0.4)	0.1 (-0.5 to 0.6)	1.0 (0.6 to 1.4)	1.4 (0.8 to 2.1)
**Education**											
No Ed.	28.3 (3.7)	28.2 (3.1)	28.7 (3.1)	27.9 (3.7)	29.1 (1.9)	28.5 (2.7)	27.8 (3.3)	30.6 (2.7)	30.1 (3.5)	27.0 (3.3)	26.1 (5.0)
Some Ed.	29.3 (2.5)	29.8 (2.2)	29.8 (2.6)	29.9 (2.2)	29.7 (2.3)	29.5 (2.9)	29.4 (2.1)	30.7 (1.4)	30.3 (3.5)	29.0 (3.3)	28.5 (1.9)
Primary	30.4 (1.8)	30.8 (1.6)	30.6 (2.4)	30.6 (1.9)	30.8 (1.6)	30.6 (1.7)	30.3 (1.8)	31.5 (2.1)	31.0 (4.2)	29.8 (3.5)	30.0 (1.6)
Secondary	31.1 (1.5)	31.1 (1.7)	31.3 (2.7)	30.1 (5.6)	31.1 (3.2)	31.3 (1.3)	30.7 (1.6)	31.9 (1.1)	32.0 (0.7)	31.1 (1.2)	30.9 (1.3)
Tertiary	31.7 (2.2)	31.1 (1.7)	31.7 (1.4)	31.1 (0.9)	31.3 (1.5)	31.6 (1.2)	31.5 (1.2)	32.0 (0.9)	31.4 (1.8)	31.0 (1.5)	31.4 (1.7)
Crude **β** (95% C.I.)	0.8 (0.7 to 0.8)	0.7 (0.6 to 0.8)	0.6 (0.5 to 0.7)	0.6 (0.4 to 0.8)	0.4 (0.3 to 0.5)	0.7 (0.6 to 0.8)	0.8 (0.7 to 0.9)	0.3 (0.2 to 0.4)	0.3 (0.2 to 0.5)	0.9 (0.8 to 1.0)	1.2 (1.0 to 1.5)
Adj. **β^3^** (95% C.I.)	0.7 (0.6 to 0.7)	0.6 (0.5 to 0.7)	0.5 (0.3 to 0.6)	0.6 (0.4 to 0.7)	0.5 (0.4 to 0.6)	0.6 (0.5 to 0.7)	0.7 (0.6 to 0.9)	0.3 (0.2 to 0.4)	0.2 (0.1 to 0.4)	0.8 (0.7 to 0.9)	0.9 (0.7 to 1.2)

DR = Dominican Republic

1. Adjusted for sex and education

2. Adjusted for age and education

3. Adjusted for age and sex

**Table 3:** Mean (SD) scores for CERAD verbal fluency (animal naming) test by demographic status and site

	**Country**										
	
	**Cuba**	**DR**	**Peru**		**Venezuela**	**Mexico**		**China**		**India**	
	Urban	Urban	Urban	Rural	Urban	Urban	Rural	Urban	Rural	Urban	Rural
**Age**											
65 - 69	18.1 (6.2)	15.0 (4.8)	19.3 (5.5)	17.1 (4.9)	20.1 (6.3)	16.9 (5.1)	14.9 (4.2)	17.3 (4.6)	16.2 (5.7)	8.9 (3.3)	10.4 (3.6)
70 - 74	16.9 (5.8)	14.2 (4.9)	18.2 (5.3)	16.1 (5.1)	18.0 (6.0)	15.7 (5.1)	14.1 (4.4)	16.8 (4.7)	15.8 (5.1)	8.6 (3.4)	10.1 (3.8)
75 - 79	15.9 (5.4)	13.7 (4.3)	16.9 (4.8)	15.2 (3.7)	16.8 (5.7)	15.2 (4.8)	13.0 (4.5)	16.3 (4.5)	14.1 (5.2)	8.4 (3.3)	9.5 (3.3)
80+	14.7 (5.6)	12.3 (4.4)	14.3 (5.1)	14.4 (5.0)	14.8 (5.8)	14.3 (5.1)	13.0 (4.6)	15.8 (4.8)	13.1 (5.0)	7.4 (2.5)	9.1 (4.4)
Crude **β** (95% C.I.)	-1.1 (-1.3 to -0.9)	-0.9 (-1.1 to -0.7)	-1.6 (-1.9 to -1.3)	-0.9 (-1.3 to -0.5)	-1.7 (-2.0 to -1.4)	-0.8 (-1.1 to -0.5)	-0.7 (-1.0 to -0.5)	-0.5 (-0.8 to -0.3)	-1.0 (-1.4 to -0.7)	-0.4 (-0.6 to -0.2)	-0.4 (-0.7 to -0.2)
Adj. **β^1^** (95% C.I.)	-0.7 (-0.9 to -0.5)	-0.8 (-1.0-0.6)	-1.4 (-1.7 to -1.2)	-0.8 (-1.2 to -0.5)	-1.4 (-1.7 to -1.1)	-0.6 (-0.9 to -0.2)	-0.7 (-0.9 to -0.4)	-0.5 (-0.8 to -0.3)	-0.7 (-1.1 to -0.4)	-0.4 (-0.6 to -0.1)	-0.4 (-0.6 to -0.2)
**Sex**											
Females	15.9 (5.6)	13.5 (4.5)	17.2 (5.3)	15.6 (4.7)	18.1 (6.2)	15.5 (5.1)	13.5 (4.3)	16.1 (4.6)	14.8 (5.1)	8.1 (3.1)	9.3 (3.7)
Males	17.7 (6.3)	14.6 (5.1)	17.8 (5.9)	16.2 (5.0)	18.7 (6.6)	16.1 (5.2)	14.4 (4.7)	17.4 (4.6)	15.9 (5.9)	9.4 (3.5)	10.7 (3.6)
Crude **β** (95% C.I.)	1.8 (1.4 to 2.3)	1.1 (0.6 to 1.6)	0.5 (-0.1 to 1.2)	0.6 (-0.2 to 1.4)	0.6 (0.0 to 1.2)	0.6 (-0.1 to 1.3)	0.9 (0.3 to 1.5)	1.2 (0.7 to 1.8)	1.1 (0.4 to 1.8)	1.3 (0.9 to 1.8)	1.4 (1.0 to 1.9)
Adj. **β^2^** (95% C.I.)	1.3 (0.9 to 1.7)	0.9 (0.4 to 1.3)	0.5 (-0.1 to 1.1)	0.5 (-0.3 to 1.3)	0.2 (-0.4 to 0.8)	0.6 (-0.1 to 1.2)	0.9 (0.4 to 1.5)	0.8 (0.3 to 1.4)	-0.1 (-0.8 to 0.7)	0.9 (0.4 to 1.3)	0.9 (0.4 to 1.3)
**Education**											
No Ed.	13.6 (4.5)	12.8 (4.3)	14.3 (4.5)	13.4 (4.1)	14.8 (5.0)	14.0 (4.4)	12.6 (4.0)	15.3 (4.4)	14.3 (5.1)	7.7 (3.1)	9.3 (3.6)
Some Ed.	13.9 (5.0)	13.4 (4.4)	14.7 (4.6)	15.7 (5.0)	16.7 (6.6)	14.5 (4.7)	14.1 (4.5)	15.2 (5.1)	14.3 (5.1)	8.9 (3.4)	10.3 (3.3)
Primary	15.6 (5.28)	14.8 (5.0)	16.1 (4.9)	16.5 (4.5)	18.8 (6.1)	16.2 (4.9)	14.8 (4.2)	17.1 (4.4)	17.5 (5.7)	10.0 (3.0)	12.0 (3.2)
Secondary	17.2 (5.5)	15.8 (5.2)	17.7 (5.6)	16.9 (6.3)	19.7 (6.0)	18.1 (4.3)	18.0 (4.5)	17.6 (4.2)	17.6 (6.4)	10.3 (3.5)	14.0 (4.1)
Tertiary	20.7 (6.4)	16.8 (5.7)	19.7 (5.7)	16.1 (6.2)	21.7 (7.3)	19.4 (6.0)	18.3 (5.0)	17.2 (5.2)	17.6 (2.7)	9.7 (3.03)	16.0 (2.8)
Crude **β** (95% C.I.)	1.9 (1.7 to 2.1)	0.7 (0.6 to 0.9)	1.4 (1.2 to 1.7)	0.8 (0.4 to 1.1)	1.2 (0.9 to 1.4)	1.1 (0.9 to 1.3)	0.9 (0.7 to 1.1)	0.5 (0.4 to 0.7)	0.9 (0.7 to 1.1)	0.5 (0.4 to 0.6)	0.9 (0.7 to 1.1)
Adj. **β^3^** (95% C.I.)	1.6 (1.4 to 1.8)	0.6 (0.4 to 0.8)	1.1 (0.8 to 1.4)	0.6 (0.3 to 0.9)	1.3 (1.1 to 1.6)	1.0 (0.8 to 1.2)	0.8 (0.6 to 1.1)	0.4 (0.2 to 0.6)	0.7 (0.5 to 1.0)	0.4 (0.3 to 0.6)	0.8 (0.5 to 1.0)

DR = Dominican Republic

1. Adjusted for sex and education

2. Adjusted for age and education

3. Adjusted for age and sex

**Table 4:** Mean (SD) scores for CERAD word list memory (immediate recall) test by demographic status and site

	**Country**										
	
	**Cuba**	**DR**	**Peru**		**Venezuela**	**Mexico**		**China**		**India**	
	Urban	Urban	Urban	Rural	Urban	Urban	Rural	Urban	Rural	Urban	Rural
**Age**											
65 - 69	17.0 (3.9)	15.07 (3.65)	16.9 (3.8)	14.0 (3.8)	16.3 (4.0)	15.0 (3.6)	13.7 (3.6)	18.7 (4.2)	15.5 (4.3)	13.5 (4.8)	8.9 (3.9)
70 - 74	16.0 (4.1)	14.37 (3.78)	15.7 (3.5)	13.2 (3.7)	15.0 (3.9)	13.5 (4.0)	12.4 (3.9)	18.0 (4.0)	14.6 (3.6)	12.3 (4.5)	7.9 (3.7)
75 - 79	15.1 (3.8)	13.43 (3.52)	14.4 (3.9)	11.9 (3.9)	14.3 (4.3)	12.1 (3.7)	11.3 (4.0)	17.5 (4.4)	13.0 (4.2)	11.6 (4.6)	8.0 (3.9)
80+	14.0 (4.1)	12.24 (3.84)	12.6 (3.6)	11.6 (3.9)	13.0 (3.9)	11.7 (4.1)	11.2 (3.8)	16.4 (4.7)	12.6 (3.6)	10.8 (4.1)	5.9 (4.2)
Crude **β** (95% C.I.)	-1.0 (-1.1 to -0.8)	-0.9 (-1.1 to -0.8)	-1.4 (-1.6 to -1.2)	-0.8 (-1.1 to -0.5)	-1.1 (-1.2 to -0.9)	-1.1 (-1.4 to -0.9)	-0.9 (-1.1 to -0.7)	-0.7 (-1.0 to -0.5)	-1.1 (-1.3 to -0.8)	-0.9 (-1.2 to -0.6)	-0.8 (-1.1 to -0.5)
Adj. **β^1^** (95% C.I.)	-0.7 (-0.8 to -0.6)	-0.9 (-1.0 to -0.7)	-1.2 (-1.4 to -1.0)	-0.7 (-0.9 to -0.4)	-0.9 (-1.1 to -0.7)	-0.9 (-1.1 to -0.6)	-0.7 (-1.0 to -0.5)	-0.7 (-0.9 to -0.4)	-0.9 (-1.2 to -0.6)	-0.9 (-1.2 to -0.6)	-0.7 (-1.0 to -0.5)
**Sex**											
Females	15.6 (4.2)	14.1 (3.8)	15.4 (4.0)	13.4 (4.1)	15.4 (4.2)	13.7 (3.9)	12.9 (3.9)	17.5 (4.1)	14.1 (3.8)	12.2 (4.5)	7.5 (4.1)
Males	15.5 (4.0)	13.5 (3.9)	14.5 (4.0)	12.3 (3.72)	14.9 (4.1)	12.5 (4.3)	11.5 (3.9)	18.3 (4.5)	14.8 (4.5)	13.0 (4.9)	8.5 (3.7)
Crude **β** (95% C.I.)	-0.1 (-0.4 to 0.2)	-0.6 (-1.0 to -0.2)	-1.0 (-1.4 to -0.5)	-1.1 (-1.8 to -0.5)	-0.5 (-0.9 to -0.1)	-1.2 (-1.7 to -0.6)	-1.3 (-1.8 to -0.8)	0.8 (0.3 to 1.3)	0.8 (0.2 to 1.3)	0.8 (0.1 to 1.4)	1.0 (0.5 to 1.5)
Adj. **β^2^** (95% C.I.)	-0.4 (-0.7 to -0.1)	-0.8 (-1.2 to -0.5)	-1.0 (-1.4 to -0.5)	-1.4 (-2.0 to -0.7)	-0.8 (-1.2 to -0.4)	-1.1 (-1.6 to -0.6)	-1.2 (-1.7 to -0.7)	0.1 (-0.4 to 0.7)	0.1 (-0.5 to 0.7)	-0.3 (-0.9 to 0.3)	-0.1 (-0.7 to 0.5)
**Education**											
No Ed.	13.4 (4.9)	13.0 (3.8)	12.3 (4.7)	10.6 (3.49)	12.1 (4.0)	11.7 (3.8)	11.3 (3.6)	16.3 (4.0)	13.8 (3.9)	11.0 (4.3)	7.2 (3.8)
Some Ed.	13.9 (4.0)	13.5 (3.7)	13.1 (3.7)	12.8 (3.91)	14.5 (4.3)	12.2 (3.8)	12.5 (3.9)	16.1 (3.7)	14.1 (4.1)	12.2 (4.1)	8.8 (3.5)
Primary	15.2 (3.9)	14.7 (3.6)	14.3 (3.9)	13.4 (3.76)	15.6 (3.9)	13.9 (3.7)	13.1 (3.7)	17.7 (4.2)	15.4 (4.3)	14.0 (4.3)	10.1 (4.1)
Secondary	16.2 (3.9)	15.7 (4.1)	15.4 (3.9)	13.5 (4.65)	15.9 (4.0)	15.1 (3.6)	14.9 (3.7)	18.9 (4.1)	17.0 (4.7)	16.0 (5.2)	10.8 (3.9)
Tertiary	17.8 (3.9)	16.1 (4.5)	16.6 (4.0)	15.1 (4.5)	16.7 (4.1)	16.8 (3.5)	17.1 (4.9)	19.7 (4.7)	14.8 (6.5)	15.5 (4.8)	12.5 (3.5)
Crude **β** (95% C.I.)	1.1 (1.0 to 1.3)	0.6 (0.5 to 0.8)	1.0 (0.8 to 1.2)	0.8 (0.5 to 1.0)	0.8 (0.6 to 0.9)	1.0 (0.8 to 1.1)	0.7 (0.5 to 0.9)	0.7 (0.5 to 0.9)	0.6 (0.4 to 0.7)	1.0 0.8 to 1.2)	0.9 (0.7 to 1.1)
Adj. **β^3^** (95% C.I.)	1.0 (0.8 to 1.1)	0.6 (0.4 to 0.7)	0.8 (0.6 to 1.0)	0.8 (0.5 to 1.0)	0.9 (0.7 to 1.1)	0.9 (0.7 to 1.1)	0.7 (0.5 to 0.9)	0.7 (0.5 to 0.8)	0.4 (0.2 to 0.6)	1.0 (0.8 to 1.2)	0.9 (0.7 to 1.1)

DR = Dominican Republic

1. Adjusted for sex and education

2. Adjusted for age and education

3. Adjusted for age and sex

**Table 5:** Mean (SD) scores for CERAD word list recall (delayed recall) test by demographic status and site

	**Country**										
	
	**Cuba**	**DR**	**Peru**		**Venezuela**	**Mexico**		**China**		**India**	
	Urban	Urban	Urban	Rural	Urban	Urban	Rural	Urban	Rural	Urban	Rural
**Age**											
65 - 69	5.7 (1.9)	4.8 (1.9)	5.7 (1.8)	4.8 (1.9)	5.6 (2.0)	5.1 (1.7)	4.8 (1.8)	7.0 (1.6)	4.2 (1.8)	4.63 (2.0)	3.2 (1.8)
70 - 74	5.3 (1.8)	4.4 (1.8)	5.2 (1.8)	4.5 (1.9)	5.2 (2.0)	4.6 (1.8)	4.1 (1.9)	6.8 (1.7)	3.9 (1.7)	4.19 (2.0)	2.9 (1.7)
75 - 79	4.9 (1.9)	4.1 (1.8)	4.6 (1.8)	3.6 (1.8)	4.8 (2.1)	4.0 (1.8)	3.6 (2.0)	6.5 (1.9)	3.3 (1.7)	3.87 (2.2)	2.8 (1.7)
80+	4.3 (1.8)	3.5 (1.9)	3.8 (1.9)	3.5 (2.0)	4.1 (2.0)	3.6 (1.8)	3.7 (1.9)	6.1 (2.2)	3.3 (1.6)	3.31 (2.1)	2.1 (1.6)
Crude **β** (95% C.I.)	-0.4 (-0.5 to -0.4)	-0.4 (-0.5 to -0.4)	-0.6 (-0.7 to -0.5)	-0.5 (-0.6 to -0.3)	-0.5 (-0.6 to -0.4)	-0.5 (-0.6 to -0.4)	-0.4 (-0.5 to -0.3)	-0.3 (-0.4 to -0.2)	-0.4 (-0.5 to -0.3)	-0.4 (-0.6 to -0.3)	-0.3 (-0.4 to -0.2)
Adj. **β^1^** (95% C.I.)	-0.3 (-0.4 to -0.3)	-0.4 (-0.5 to -0.3)	-0.6 (-0.6 to -0.5)	-0.4 (-0.5 to -0.3)	-0.4 (-0.5 to -0.3)	-0.4 (-0.5 to -0.3)	-0.3 (-0.5 to -0.2)	-0.3 (-0.4 to -0.2)	-0.3 (-0.4 to -0.2)	-0.4 (-0.6 to -0.3)	-0.3 (-0.4 to -0.2)
**Sex**											
Females	5.1 (1.9)	4.4 (1.9)	5.1 (2.0)	4.4 (2.1)	5.3 (2.0)	4.6 (1.9)	4.3 (2.0)	6.6 (1.8)	3.7 (1.7)	4.2 (1.9)	2.7 (1.7)
Males	5.0 (1.9)	4.0 (1.8)	4.6 (1.9)	3.9 (1.9)	4.9 (2.0)	4.1 (1.9)	3.8 (1.9)	6.8 (1.9)	4.0 (1.8)	4.3 (2.2)	3.1 (1.8)
Crude **β** (95% C.I.)	-0.1 (-0.3 to 0.0)	-0.3 (-0.5 to -0.2)	-0.5 (-0.7 to -0.2)	-0.5 (-0.8 to -0.2)	-0.3 (-0.5 to -0.1)	-0.5 (-0.8 to -0.3)	-0.5 (-0.8 to -0.3)	0.3 (0.1 to 0.5)	0.2 (0.0 to 0.5)	0.2 (-0.1 to 0.4)	0.4 (0.2 to 0.6)
Adj. **β^2^** (95% C.I.)	-0.2 (-0.4 to -0.1)	-0.4 (-0.6 to -0.3)	-0.4 (-0.7 to -0.2)	-0.6 (-0.9 to -0.3)	-0.4 (-0.6 to -0.2)	-0.5 (-0.7 to -0.21)	-0.5 (-0.7 to -0.2)	0.0 (-0.2 to 0.3)	0.0 (-0.3 to 0.2)	-0.2 (-0.5 to 0.04)	0.0 (-0.3 to 0.2)
**Education**											
No Ed.	4.2 (2.1)	4.0 (2.0)	4.0 (2.0)	3.1 (1.8)	4.1 (1.7)	3.9 (1.9)	3.7 (2.1)	6.2 (1.9)	3.6 (1.7)	3.7 (2.0)	2.6 (1.6)
Some Ed.	4.4 (1.9)	4.1 (1.8)	4.3 (1.9)	4.1 (2.2)	4.9 (2.1)	4.0 (1.8)	4.2 (1.9)	5.7 (1.8)	4.0 (1.7)	3.9 (1.8)	3.2 (1.8)
Primary	5.0 (1.8)	4.5 (1.7)	4.5 (1.9)	4.4 (1.8)	5.3 (2.0)	4.7 (1.8)	4.5 (1.8)	6.8 (1.7)	4.2 (1.8)	4.8 (2.0)	3.7 (1.8)
Secondary	5.3 (1.8)	4.9 (1.9)	5.1 (1.8)	4.8 (1.7)	5.3 (1.9)	5.3 (1.9)	5.1 (2.2)	7.1 (1.7)	4.6 (1.9)	5.5(2.1)	4.1 (1.7)
Tertiary	6.0 (2.0)	5.0 (2.1)	5.4 (2.0)	5.4 (1.7)	5.8 (2.1)	5.4 (1.7)	5.8 (1.6)	7.3 (1.9)	3.8 (2.7)	5.3 (1.6	4.0 (1.4)
Crude **β** (95% C.I.)	0.5 (0.4 to 0.5)	0.2 (0.1 to 0.3)	0.4 (0.3 to 0.5)	0.4 (0.2 to 0.5)	0.3 (0.2 to 0.4)	0.3 (0.3 to 0.4)	0.3 (0.2 to 0.4)	0.3 (0.2 to 0.3)	0.2 (0.1 to 0.3)	0.3 (0.3 to 4.0)	0.3 (0.2 to 0.4)
Adj. **β^3^** (95% C.I.)	0.4 (0.3 to 0.5)	0.2 (0.1 to 0.3)	0.3 (0.2 to 0.4)	0.4 (0.2 to 0.5)	0.3 (0.2 to 0.4)	0.3 (0.2 to 0.4)	0.3 (0.2 to 0.4)	0.2 (0.2 to 0.3)	0.2 (0.1 to 0.2)	0.4 (0.3 to 0.4)	0.3 (0.2 to 0.4)

DR = Dominican Republic

1. Adjusted for sex and education

2. Adjusted for age and education

3. Adjusted for age and sex

Tests for interaction indicated that the effects of age, sex and education on cognitive test performance were each significantly modified by site for all four cognitive tests. However, the effects were uniformly very modest in size, generally accounting for between 0.1% and 0.3% of the overall variance. The two largest interaction effects were those for verbal fluency between site and age (0.6% of the variance), and site and education (0.5%).

Table 6 summarises the independent effects of age, sex, education and site on cognitive test performance. Site accounted for the highest proportion of variance for all four scores followed by education and then age, except for WLR where the effect of age was stronger than education. The contribution of sex to the models was uniformly low. Most of the effect of site could be more parsimoniously accounted for by region (Latin America versus (a) China, and (b) India) and, to a lesser extent, rural versus urban location (with marginally poorer performance on WLM and WLR in rural compared with urban settings). Controlling for age, education, sex and rural/urban location, performance on all cognitive tests was best among Chinese participants, intermediate among Latin American participants, and worst among Indian participants. Chinese participants scored one point more and Indian participants one and a half points less on the COGSCORE than did participants in Latin American sites. Indian participants generated nearly six fewer animals on verbal fluency than did participants in China and Latin America. Compared with Latin American participants, Chinese participants remembered on average nearly three more words out of 30 on WLM and one more word out of 10 on delayed WLR. Indian participants, on the other hand, recalled on average two and a half words fewer on WLM and half a word fewer on WLR.

**Table 6:** The independent effects of age, education, sex and site on the four cognitive assessments, further decomposing the effect of site into region and rural/urban location

	The independent, mutually adjusted effects (eta^2 ^%) of age, education, sex and site on cognitive test performance				Unpacking 'site' - the independent effects (eta^2 ^%) of region and rural/urban location		The effect of region, adjusting for age, education, sex and rural/urban location (95% CI)			
Cognitive test	Age	Education	Sex	Site	Region	Rural/Urban	Parameter	Latin America	China	India

Global function (COGSCORE)	3.3%	7.0%	0.4%	7.6%	6.8%	0.5%	Adjusted mean	30.0 (29.9, 30.1)	31.1 (31.0, 31.2)	28.5 (28.3, 28.6)
							Mean difference	Reference	1.1 (0.9, 1.2)	-1.6 (-1.7, -1.4)
Verbal Fluency	3.1%	5.1%	0.4%	15.8%	12.2%	0.0%	Adjusted mean	16.0 (15.9, 16.1)	16.3 (16.1, 16.5)	10.2 (10.0, 10.5)
							Mean difference	Reference	0.3 (0.0, 0.5)	-5.8 (-6.1, -5.5)
Word list memory	4.9%	6.5%	0.5%	16.0%	11.6%	4.1%	Adjusted mean	13.5 (13.4, 13.6)	16.3 (16.1, 16.5)	11.0 (10.7, 11.2)
							Mean difference	Reference	2.8 (2.6, 3.0)	-2.5 (-2.8, -2.3)
Word list recall	4.7%	4.0%	0.6%	11.6%	5.1%	3.5%	Adjusted mean	4.3 (4.3, 4.3)	5.4 (5.3, 5.5)	3.8 (3.7, 3.9)
							Mean difference	Reference	1.1 (1.0, 1.2)	-0.5 (-0.6, -0.4)

## Discussion

We have provided normative data by age group, sex and educational level for widely used neuropsychological tests of global cognitive function, verbal fluency and immediate and delayed word recall in seven low or middle income countries. People with any degrees of dementia, including questionable dementia, were excluded. These norms have been rigorously generated applying a standardized testing procedure amongst representative community-dwelling samples. To our knowledge this is the largest study to date on neuropsychological tests norms and the first to present direct comparisons between so many culturally diverse countries.

With the exception of rural India, our norms for CERAD WLM and WLR are well aligned with those previously reported from affluent western countries. [4,22-24]. Our norms for CERAD VF are comparable to previously determined norms from both Europe and North America countries [22,23,25,26] and from Latin America [27-29]. We found that older age and lower educational level corresponded to poorer performances in all four tests and across all sites. The influences of age and educational level on test performances were large, and consistent in size and direction with other normative data investigations from western countries [23]. Sex had a much weaker influence and can probably be safely ignored when constructing reference norms. Likewise, while the site by age, education and sex interactions were statistically significant for all cognitive tests, these were very modest effects, and the beta coefficients (Tables 2, 3, 4 and 5) are remarkable mainly for their consistency across sites.

There was a considerable residual effect of site upon cognitive test performance, not accounted for by compositional differences between samples in the distribution of age and education. Further analyses clarified that the between-site difference was most parsimoniously accounted for by the effect of region, with smaller effects of rural versus urban location evident for the two memory tests. We should still be cautious about attributing the effect of region to that of language and culture. First, other compositional differences not directly linked to culture *per se*, but relevant to cognitive performance and differently distributed across sites, may not have not been taken into consideration in our analyses. One such effect may be the quality and nature of education received that may not be adequately summarised in terms of level of education [30]. Second, while we have included a wide variety of Latin American and Hispanic Caribbean countries and shown fairly consistent norms between them, the norms derived from the Tamil speaking Indians in Tamil Nadu, and the Mandarin-speaking Chinese in and around Beijing clearly cannot be generalised to the vast and diverse populations of India and China as a whole.

By design, the two cognitive tests included in the 10/66 dementia diagnosis, the CSI 'D' COGSCORE and the CERAD WLR, were those that showed the smallest cultural influences and the most robust cross-cultural discriminating properties [9]. This finding has now been, in part, replicated in the population-based phase of our study and is reassuring with respect to the cross-cultural validity of that diagnosis. However, in the light of the findings with respect to other tests, it may be necessary in the future to use region-specific norms for the identification of impairment in immediate recall or verbal fluency for the identification of those meeting cognitive impairment criteria (1.5 standard deviations below the age- and education-specific norms for those with no dementia) for DSM-IV dementia [31], and amnestic and non-amnestic mild cognitive impairment. The general effect of such a change would be to lower still further the already negligible prevalence of DSM-IV dementia in Indian sites, and to increase slightly the prevalence of DSM-IV dementia in Chinese sites.

## Conclusion

Cognitive assessment is a basic element for dementia diagnosis. Age- and education-specific norms are required for this purpose, while the effect of gender can probably be ignored. The basis of cultural effects is poorly understood, but our findings serve to emphasise that normative data may not be safely generalised from one population to another with quite different characteristics. The minimal effects of region on COGSCORE and WLR are reassuring with respect to the cross-cultural validity of the 10/66 dementia diagnosis, which uses only these elements of the 10/66 battery.

## Competing interests

The 10/66 Dementia Research Group works closely with Alzheimer's Disease International (ADI), the non-profit federation of 77 Alzheimer associations around the world. ADI is committed to strengthening Alzheimer associations worldwide, raising awareness regarding dementia and Alzheimer's Disease and advocating for more and better services for people with dementia and their caregivers. ADI is supported in part by grants from GlaxoSmithKline, Novartis, Lundbeck, Pfizer and Eisai.

## Authors' contributions

All of the authors worked collectively to develop the protocols and methods described in this paper. MP led the research group and CF acted as research coordinator. JLR (Cuba), DA (Dominican Republic), MG (Peru), AS (Venezuela), ALS (Mexico), KSJ (Vellore, India), JDW (Chennai, India) and YH (China) were principal investigators responsible for the field work in their respective countries. ALS, EA and RS prepared the first draft and carried out the analyses of this manuscript. MP carried out a final revision for the first submission and RS revised the second submission in response to reviewers' comments. Other authors reviewed the manuscript, provided additional comments and contributions. All authors read and approved the final version of the manuscript.

## Pre-publication history

The pre-publication history for this paper can be accessed here:


